# Extractable Cr(VI) Hotspots in the Defor Petrila Tailings Dump, Romania: A Redox-Based Hazard Screening Approach

**DOI:** 10.3390/toxics14060479

**Published:** 2026-05-30

**Authors:** Mădălina F. Ioniță, Emilia C. Dunca, Sorin M. Radu, Sabin I. Irimie

**Affiliations:** 1 Department of Environmental Engineering and Geology, Faculty of Mining, University of Petrosani, 332006 Petrosani, Romania; madalina-flaviaionita@upet.ro; 2 Department of Mechanical, Industrial and Transport Engineering, University of Petrosani, 332006 Petrosani, Romania; 3 Department of Management and Industrial Engineering, Faculty of Mining, University of Petrosani, 332006 Petrosani, Romania

**Keywords:** chromium(VI), mine tailings, redox conditions, manganese oxides, hazard screening, contaminated soils, Jiu Valley, chromium speciation

## Abstract

Chromium-related hazard in mine wastes depends strongly on oxidation state, with hexavalent chromium [Cr(VI)] representing the most mobile and toxicologically relevant chromium form. Abandoned tailings dumps can develop sharp pH and redox gradients that favour either Cr(VI) persistence or attenuation, yet field-based evidence from Eastern European post-mining sites remains limited. This study evaluates the Defor Petrila tailings dump, Jiu Valley, Romania, as a first-tier environmental hazard-screening case study based on repeated monitoring performed during 2022–2024 at twelve permanent sampling points and two local operational control samples. Field pH and redox potential (Eh), moisture, organic matter, acid-extractable Mn and Fe, pseudo-total Cr, and method-defined extractable Cr(VI) were determined. Here, pseudo-total Cr refers to chromium released by microwave-assisted acid digestion and does not represent complete decomposition of the silicate matrix, while extractable Cr(VI) refers to the operationally defined fraction obtained by alkaline extraction. In addition, a conservative redox-based prioritisation score (R_redox_) was applied only as an internal ranking layer to identify sectors where Cr(VI) is more likely to persist. The upper dump sector (P1–P4) was alkaline (pH 7.5–8.2), strongly oxidising (+280 to +412 mV), and enriched in Mn and Fe, whereas the lower sector (P9–P12) was wetter, slightly acidic to near-neutral, and reducing (−59 to −10 mV). Extractable Cr(VI) reached 18.7 mg kg^−1^ at P2 in 2024, while both control samples remained below the quantification limit. Exploratory repeated-site statistics, sector-based comparison, and correlation analysis supported a coherent association between Eh, Mn enrichment, and extractable Cr(VI), but these relationships are interpreted as spatially structured screening evidence rather than proof of a single mineralogical oxidation pathway. No direct exposure, leachability, bioaccessibility, ecotoxicity, airborne dust, water, vegetation, or biomonitoring measurements were included; therefore, the results identify priority zones for confirmatory toxicological and exposure-based assessment, not receptor-specific risk estimates. This study demonstrates that combining chromium speciation with field redox zonation can support conservative monitoring prioritisation at abandoned mine-waste sites where the toxic form of chromium may remain environmentally active.

## 1. Introduction

Chromium is a redox-sensitive element whose environmental and toxicological behaviour depends strongly on valence state [1,2,3,4,5,6,7]. In contaminated soils and mine wastes, trivalent chromium is generally less mobile and less bioavailable, whereas hexavalent chromium is more soluble, more mobile, and toxicologically more relevant because it can enter biological systems as chromate oxyanions. The distinction is particularly important at abandoned mining sites, where weathering can progressively transform relatively stable mineral-bound chromium into environmentally available Cr(VI) near the surface.

The formation and persistence of Cr(VI) in soils and tailings are controlled by coupled geochemical processes involving pH, redox potential, moisture regime, reactive mineral phases, and organic matter [8,9,10,11,12,13,14,15]. Oxidation of Cr(III) is favoured in alkaline and oxidising environments, especially where Mn-bearing phases act as effective oxidants, while reducing and organic-rich compartments promote Cr(VI) attenuation or back-reduction [11,16]. These mechanisms make chromium-contaminated mine wastes dynamic systems in which toxic hazard is not controlled only by total chromium concentration but also by the local geochemical architecture that determines whether Cr(VI) can form and remain stable.

Tailings dumps are, therefore, relevant not only as sources of potentially toxic elements but also as reactive surfaces where toxic species can be generated, retained, or released [17,18,19]. Near-surface oxidised materials may favour dust generation during dry periods, while downslope compartments may receive dissolved or particulate chromium through runoff, infiltration, and internal redistribution [9,10,20,21,22,23,24]. For a toxicological interpretation, the key issue is not merely whether chromium is present, but whether site conditions support persistence of the toxic form and create plausible transfer routes. Nevertheless, pathway plausibility should be distinguished from quantified exposure or receptor-specific risk, which require additional measurements such as leachability, bioaccessibility, airborne dust, water, vegetation, or biomonitoring data [21,22,23,25,26].

The Jiu Valley is a long-established mining region in Romania that still contains numerous post-closure waste deposits requiring monitoring, stabilisation, and environmentally sound reuse. Previous work at the Defor Petrila tailings dump documented broader soil contamination patterns and the presence of geochemical contrasts linked to site morphology, while national studies have highlighted the continuing remediation challenges posed by Romanian mining areas [27,28]. However, field evidence focused specifically on Cr(VI) and its redox controls, and its toxicological significance remains limited for this region.

Against this background, the present study was designed as a field-based geochemical assessment with explicit toxicological relevance, focused on the chromium fraction of greatest concern for mobility and biological reactivity. The objectives were to (i) characterise the spatial and temporal distribution of pseudo-total chromium and method-defined extractable Cr(VI) in the Defor Petrila tailings dump; (ii) identify the pH-Eh-Mn-Fe conditions associated with redox-controlled Cr(VI) hotspots; and (iii) test a transparent redox-based prioritisation score (R_redox_) for ranking sectors where Cr(VI) persistence and environmental transfer potential warrant confirmatory investigation. The working hypothesis was that the upper, better-aerated sector of the dump would promote sustained Cr(VI) occurrence, whereas the lower, wetter sector would behave as a comparatively reducing attenuation compartment.

The specific contribution of this manuscript is the integration of extractable Cr(VI), field redox zonation, and an operational prioritisation score to identify internally differentiated chromium hazard compartments within the Defor Petrila tailings dump. Rather than treating the deposit as a homogeneous contaminated body, this study frames Cr(VI) occurrence as a spatially structured hazard controlled by local redox architecture. The manuscript, therefore, addresses a first-tier question—where extractable Cr(VI) is most likely to persist under field conditions—without claiming to quantify receptor-specific exposure, ecological toxicity or human-health risk.

## 2. Materials and Methods

### 2.1. Study Area and Toxicological Relevance

The Defor Petrila mine tailings dump is located in the eastern part of the Jiu Valley, Romania, at approximately 45.448° N and 23.397° E, between 650 and 830 m above sea level. The deposit is an abandoned mining-waste body with clear topographic differentiation between an upper plateau, transitional slopes, and a lower accumulation sector. This morphology produces short-distance contrasts in aeration, drainage, surface exposure, moisture retention, and organic matter accumulation, all of which are relevant to chromium redox cycling. Previous site-scale assessment showed that the deposit contains multiple potentially toxic elements and should be regarded as a post-mining source area requiring continued environmental surveillance [27].

**Figure 1:**
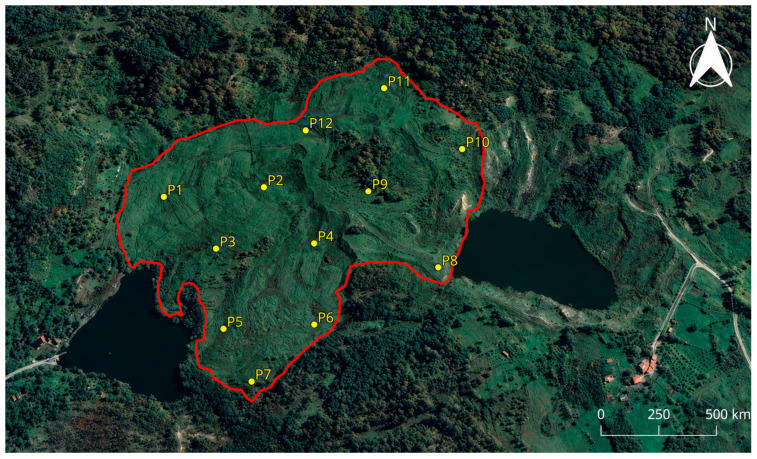
Study area of the Defor Petrila tailings dump, showing the monitored sampling points (P1–P12) and the delineated boundary of the waste deposit.

From the perspective of potential toxic hazard, the site is relevant because it combines exposed near-surface waste, sharp redox gradients, and spatial proximity to aquatic receptors and residential land uses. Such a configuration creates plausible, but not quantified, transfer scenarios through dust generation, runoff, infiltration, and downslope redistribution. The present study, therefore, focused on the 0–20 cm layer, where contact potential and oxidative weathering are greatest. Because no direct exposure, bioaccessibility, leachability, dust, water, vegetation, ecotoxicity, or biomonitoring measurements were included, the site evaluation is explicitly framed as first-tier environmental hazard screening and monitoring prioritisation rather than as a complete human-health or ecological risk assessment [17,26]. The location of the Defor Petrila tailings dump, the delineated boundary of the waste deposit, and the distribution of the monitored sampling points are shown in Figure 1.

### 2.2. Sampling Strategy and Sample Preparation

Field campaigns were conducted in September 2022, August 2023, and July 2024. Twelve permanent sampling points (P1–P12) were distributed along the main geomorphological gradient of the dump and were interpreted as three operational sectors: an upper oxidised sector (P1–P4), a transitional sector (P5–P8), and a lower accumulation/attenuation sector (P9–P12). Control 1 and Control 2 were positioned on adjacent non-tailings ground outside the mapped dump boundary and outside areas showing visible waste deposition, erosion channels, or recent material redistribution. Their purpose was to provide a local operational contrast to the tailings-affected materials rather than to define a regional background population. These samples were, therefore, used to verify whether the Cr(VI) signal observed in the dump body was also present in nearby non-tailings soils under comparable local environmental conditions.

At each monitoring point, three field subsamples were taken from the 0–20 cm layer using a stainless-steel Edelman auger (Eijkelkamp Soil & Water, Giesbeek, The Netherlands) and combined into one representative composite sample. The composite design reduced small-scale heterogeneity but did not produce independent laboratory replicates; therefore, the values reported in Table 1, Table 2, Table 3 and Table 4 represent site-year composite results.

Reported values were rounded to the precision shown in the tables after analytical processing. Because field subsamples were composited before laboratory analysis, the dataset is suitable for repeated spatial screening and sector-level comparison, but it was not designed to estimate within-point microscale variance or to support replicate-level inferential statistics. This reporting choice prevents pseudo-replication and clarifies that the strongest evidence in the manuscript is the repeated upper-to-lower contrast in redox state and Cr(VI) occurrence.

The collected material was transported to the laboratory in polyethylene bags, air-dried at room temperature, disaggregated, and sieved to <2 mm. Samples were transported to the laboratory as soon as possible after collection, protected from direct sunlight and excessive heating. During handling, the same preparation sequence was applied to all samples in order to minimise method-induced variability among years. Because Cr(VI) in solid matrices can be affected by redox transformations during storage and preparation, the results are interpreted as operationally defined extractable concentrations rather than as absolute in situ Cr(VI) speciation. Representative subsamples intended for elemental analysis were additionally homogenised by fine grinding. The monitoring design was intended to capture both the spatial structure of the dump and year-to-year variability in parameters known to influence chromium speciation.

### 2.3. Field and Laboratory Determinations

Soil/tailings pH and redox potential (Eh) were measured in situ using a WTW Multi 3630 IDS multiparameter instrument equipped with field electrodes (Xylem Analytics Germany GmbH, Weilheim, Germany). The pH electrode was calibrated before each field measurement session using standard pH 4.00, 7.00, and 10.00 buffer solutions (Merck KGaA, Darmstadt, Germany), covering the slightly acidic to alkaline range observed in the investigated materials. The redox electrode response was checked before field use according to the manufacturer’s recommendations, and the electrodes were rinsed with deionised water between measurements to minimise carry-over. Eh values are reported consistently as field-measured potentials and are used for within-site redox contrast and screening interpretation; no thermodynamic speciation modelling or Pourbaix-level equilibrium calculation was attempted. Moisture content was determined gravimetrically after oven drying at 105 °C for 24 h using a laboratory drying oven (Memmert GmbH + Co. KG, Schwabach, Germany), and organic matter (OM) was determined by loss on ignition at 550 °C for 4 h using a muffle furnace (Nabertherm GmbH, Lilienthal, Germany). Mn, Fe, and Cr were quantified after microwave-assisted acid digestion using a microwave digestion system (Milestone Srl, Sorisole, Italy) following U.S. EPA Method 3051A and instrumental determination by flame atomic absorption spectrometry using a flame atomic absorption spectrometer (Analytik Jena GmbH, Jena, Germany) [29,30]. Because EPA 3051A is an acid-digestion procedure and not a complete HF-based decomposition of the silicate matrix, the elemental results are interpreted as pseudo-total or acid-extractable concentrations.

All reagents were of analytical grade, and calibration solutions, procedural blanks, repeated calibration checks, and laboratory control checks were used throughout the analytical sequence. Analytical results are reported on a dry-mass basis and are interpreted as screening-level measurements within an operationally defined extraction framework. Moisture and OM are used as contextual descriptors of site conditions rather than as independent annual trend variables. The aim of the Mn and Fe determinations was not only descriptive, but also interpretive, because these elements are linked to redox-active mineral phases capable of affecting chromium oxidation, sorption, and stabilisation [8,11,12,14]. However, in the absence of direct mineralogical evidence, Mn and Fe concentrations are used here as indirect geochemical indicators rather than as proof of specific Mn- or Fe-bearing mineral phases.

### 2.4. Determination of Pseudo-Total Chromium and Extractable Cr(VI)

Pseudo-total chromium was determined on finely homogenised subsamples after microwave-assisted acid digestion using a microwave digestion system (Milestone Srl, Sorisole, Italy) following U.S. EPA Method 3051A [30]. Because this digestion procedure does not ensure complete decomposition of resistant silicate phases, chromium measured after digestion is reported as pseudo-total Cr rather than as absolute total Cr. Extractable Cr(VI) was determined on a separate air-dried and <2 mm sieved subsample as an operationally defined fraction, using alkaline extraction followed by colourimetric determination with 1,5-diphenylcarbazide (Merck KGaA, Darmstadt, Germany). The extraction followed the operational principles of U.S. EPA Method 3060A [29]. Briefly, 2.5 g of homogenised soil/tailings material was mixed with 50 mL of alkaline extracting solution consisting of 0.28 M Na_2_CO_3_ (Merck KGaA, Darmstadt, Germany) and 0.5 M NaOH (Merck KGaA, Darmstadt, Germany), corresponding to a solid-to-liquid ratio of 1:20 (*w*/*v*). The suspensions were stirred, heated at 90–95 °C for 60 min, cooled to room temperature, quantitatively transferred, and filtered through 0.45 µm membrane filters (Merck Millipore, Burlington, MA, USA) before colour development.

The filtered alkaline extracts were analysed according to the colourimetric principle of U.S. EPA Method 7196A [31]. For colour development, an aliquot of the extract was acidified with sulfuric acid (Merck KGaA, Darmstadt, Germany) according to the analytical conditions required for the Cr(VI)–diphenylcarbazide reaction, reacted with 1,5-diphenylcarbazide, and measured spectrophotometrically at 540 nm using a 1 cm optical path length and a UV–Vis spectrophotometer (Analytik Jena GmbH, Jena, Germany). Procedural blanks, calibration standards, repeated calibration checks, and laboratory control checks were included in the analytical sequence to monitor contamination and instrumental response. Procedural blanks were processed through the same extraction and colour-development sequence as the samples and were used to check possible Cr(VI) contamination introduced during digestion, filtration, reagent addition, or spectrophotometric measurement. Calibration verification standards were analysed repeatedly during the analytical sequence to confirm instrumental stability. Samples and alkaline extracts were handled consistently across the three monitoring years to minimise method-induced variability.

Independently documented matrix-specific spike recovery data and certified reference material results were not available for the present reporting dataset; therefore, Cr(VI) results are interpreted conservatively as method-defined extractable concentrations, and no recovery correction was applied. Although this represents an analytical limitation, the hotspot interpretation is unlikely to be driven by analytical noise because the upper-sector Cr(VI) concentrations were one to two orders of magnitude above the dry-mass LOQ and were spatially coherent with independent field variables, including pH, Eh, and Mn enrichment. This limitation is explicitly incorporated into the uncertainty framework because Cr(VI) in solid mining-impacted matrices may be affected by extraction conditions, pH adjustment, matrix interferences, and redox transformations during handling [32,33].

For the extract solution, the analytical limit of detection and the limit of quantification were 0.002 mg/L and 0.006 mg/L, respectively. Under the extraction conditions described above, these correspond approximately to 0.04 mg/kg and 0.12 mg/kg dry material, before any additional dilution correction. Cr(VI) concentrations were converted to mg/kg dry material using the extracted soil mass, final extract volume, dilution factor, and moisture-corrected dry mass. Results below the quantification limit were not converted into fixed numerical values for ratio or index calculations; they were reported as below LOQ in order to avoid overinterpretation of low-level background signals. This reporting approach strengthens the screening interpretation by preventing artificial inflation of Cr(VI)/pseudo-total Cr fractions at low concentrations.

Analytical confidence was strengthened through a conservative reporting framework. First, Cr(VI) was reported strictly as a method-defined extractable fraction, so the manuscript does not claim absolute solid-phase Cr(VI) speciation [31,32,33]. Second, procedural blanks, calibration standards, repeated calibration checks, and laboratory control checks were used to verify contamination control and instrumental response during the analytical sequence. Third, values below the quantification limit were reported as below-threshold observations and were not converted into fixed numerical values for ratio-based mechanistic interpretation. Fourth, the highest hotspot concentrations were far above the operational LOQ; for example, the 2024 concentrations at P1–P4 were approximately 97–156 times higher than the dry-mass LOQ of 0.12 mg/kg. Therefore, the identification of the upper-sector hotspot does not depend on signals close to the detection boundary, even though exact absolute Cr(VI) concentrations should still be regarded as operationally defined.

### 2.5. Redox-Based Prioritisation Score for Hazard Screening

To translate chromium speciation data into a reproducible site-screening framework, an operational redox-based prioritisation score, denoted R_redox_, was calculated by integrating the extractable Cr(VI)/pseudo-total Cr fraction with pH- and Eh-based weighting factors. The score was designed only for within-site prioritisation of redox domains where Cr(VI) is more likely to persist; it is not a regulatory risk metric, not a toxicological benchmark, and not a substitute for direct toxicity, leaching, bioaccessibility, mineralogical, or exposure measurements. Its added value is that it summarises the measured occurrence of extractable Cr(VI) together with the field conditions that support persistence, thereby helping to rank monitoring locations for confirmatory testing and management attention. The score was calculated using the following expression:

(1) $$R_{redox}=F_{Cr(VI)}\times \left[\alpha_{pH}+\beta_{Eh}\right]$$

where

(2) $$F_{Cr(VI)}=\frac{C_{Cr(VI),ext}}{C_{Cr,total}}$$

F_Cr(VI)_ was defined as the ratio between extractable C_r(VI)_ and pseudo-total Cr measured in the same site-year sample. Under internally consistent analytical conditions, F_Cr(VI)_ is expected to vary between 0 and 1. Values below the Cr(VI) quantification limit were not used to infer low-level fractions. If an apparent F_Cr(VI)_ value were to exceed 1, it would be treated as analytically inconsistent and excluded from R_redox_ interpretation, because extractable Cr(VI) cannot reasonably exceed the operationally measured pseudo-total Cr pool under the same reporting basis.

The pH and Eh weighting factors used in the R_redox_ calculation are presented in Table 1. They were defined as transparent, expert-based screening weights reflecting the main redox domains observed at the site.

These thresholds were selected to reflect field-relevant geochemical domains observed at the site: acidic to near-neutral or organic-rich reducing compartments, transitional compartments, and alkaline oxidising compartments in which Cr(VI) persistence is expected to be favoured [8,11,12,14,15]. The pH and Eh terms were combined additively because they represent complementary constraints on Cr(VI) stability rather than independent probability terms; a multiplicative formulation was avoided because it would imply a calibrated interaction strength that the present screening dataset cannot support. The weights were not statistically fitted to the dataset and should be regarded as expert-defined, transparent, and site-specific.

For interpretation, three operational screening classes were used: low redox-driven Cr(VI) priority for R_redox_ < 0.05, moderate priority for 0.05 ≤ R_redox_ < 0.10, and high priority for R_redox_ ≥ 0.10. These classes are internal ranking categories, not regulatory thresholds, not toxicological benchmark values, and not health-risk quotients. They are used only to rank monitoring points within the Defor Petrila dump and to identify sectors where denser sampling, leachability testing, oral and inhalation bioaccessibility assessment, mineralogical confirmation, ecotoxicity testing, or receptor-specific exposure analysis should be prioritised. The proposed classes must not be transferred to other mining areas without recalibration against local geochemical, climatic, mineralogical, and exposure conditions.

R_redox_ is, therefore, used as a first-stage prioritisation score rather than as a universal screening index. Values calculated for samples with Cr(VI) below the quantification limit were retained only as below-threshold estimates and were not used to infer low-level mechanistic differences. The score is intended to complement, not replace, direct measurements of Cr(VI), leachability, bioaccessibility, ecotoxicity, exposure pathways, and mineralogy [21,22,23,24,25].

As an additional robustness check, the categorical interpretation of R_redox_ was examined qualitatively by varying both α_(pH)_ and β_(Eh)_ by ±0.1 within their respective classes. This check was not used to recalibrate the index, but to identify whether the hotspot interpretation depended on marginal weighting choices. The persistent high-priority interpretation of the upper sector was considered robust only where classification remained stable or where adjacent points formed a coherent spatial cluster.

Importantly, R_redox_ did not create a hotspot pattern that was absent from the raw dataset. The same upper-sector priority zone was already evident from measured extractable Cr(VI), Cr(VI)/pseudo-total Cr fractions, alkaline pH, positive Eh, Mn enrichment, and the spatial clustering of P1–P4. Therefore, R_redox_ should be interpreted as a decision-support synthesis and communication tool, not as the primary evidence for hazard identification.

### 2.6. Statistical and Sector-Based Analysis

Normality of the main variables was checked using the Shapiro–Wilk test, but the small, spatially structured monitoring design was not treated as a basis for strong distributional inference. Because the same permanent points were monitored across three years and the three field subsamples were composited before analysis, the dataset was treated as a repeated-site monitoring design rather than as independent annual samples. The inferential dataset, therefore, consisted of 12 complete site blocks (P1–P12) observed in 2022, 2023, and 2024; the two control samples were excluded from inferential testing and retained only as operational background references. Interannual differences were assessed using the Friedman repeated-measures test. Associations among pH, Eh, Mn, Fe, pseudo-total Cr, and extractable Cr(VI) were evaluated using Spearman rank correlations calculated on the pooled P1–P12 monitoring dataset (*n* = 36 observations). Because repeated observations and the strong upper-to-lower geomorphological gradient reduce statistical independence, correlation results are interpreted as spatially structured associations supporting the screening model, not as independent causal proof.

No predictive regression model was fitted because the dataset size, compositing strategy, and spatial structure do not support independent mechanistic modelling. As a complementary robustness check, the dataset was also interpreted by operational sector: upper oxidised sector (P1–P4), transitional sector (P5–P8), and lower accumulation/attenuation sector (P9–P12). This sector-based reading was used to verify whether the main interpretation persisted without relying on correlation coefficients or the numerical R_redox_ classes. The statistical tests were used only to evaluate coherence between field observations and the proposed screening interpretation. All numerical data were first organised and checked in Microsoft Excel 2021 (Microsoft Corporation, Redmond, WA, USA). Statistical analyses were then performed using R software version 4.4.2 (R Foundation for Statistical Computing, Vienna, Austria), while graphical representations of the monitored physicochemical and geochemical parameters were prepared using Microsoft Excel 2021. Spatial mapping and layout preparation for Figure 1 were carried out using QGIS version 3.34.15 LTR (QGIS Development Team, Open Source Geospatial Foundation Project).

### 2.7. Interpretive Safeguards and Uncertainty Control

Several safeguards were applied to reduce overinterpretation of the dataset. First, all metal fractions are described using operational terminology: pseudo-total Cr, acid-extractable Mn and Fe, and method-defined extractable Cr(VI). Second, non-detects and values below the quantification limit were treated conservatively and were not converted into fixed numerical estimates for mechanistic interpretation. Third, the two control samples were retained as local operational reference materials only and were not treated as a statistically sufficient regional background dataset. Fourth, the available QA/QC information supports conservative screening-level reporting but is not equivalent to full interlaboratory validation of Cr(VI) speciation in a complex tailings matrix [31,32,33].

To make the inferential boundary explicit, conclusions were organised according to three evidence tiers. Tier 1 evidence consists of directly measured operational data, including pH, Eh, moisture, organic matter, pseudo-total Cr, acid-extractable Mn and Fe, and extractable Cr(VI). Tier 2 evidence consists of co-located and repeated spatial associations among these variables across permanent monitoring points and operational sectors. Tier 3 evidence consists of inferred exposure pathways and mineralogical mechanisms, which are discussed as plausible interpretations but not treated as demonstrated outcomes. All main conclusions are based on Tier 1 and Tier 2 evidence; Tier 3 elements are used only to define priorities for future validation.

Fifth, R_redox_ was interpreted only as a ranking tool for prioritising monitoring locations within the same tailings body. It was not used to infer dose, exposure probability, ecological toxicity, or human-health risk [21,22,23,24,25]. Finally, statistical associations were evaluated together with spatial coherence, field conditions, and known chromium redox behaviour, so that correlation results were not treated as stand-alone evidence of causality. These safeguards define the manuscript as a conservative screening study and reduce the risk of extending the conclusions beyond the available measurements.

## 3. Results

### 3.1. Physicochemical Heterogeneity and Redox Zonation

**Figure 2:**
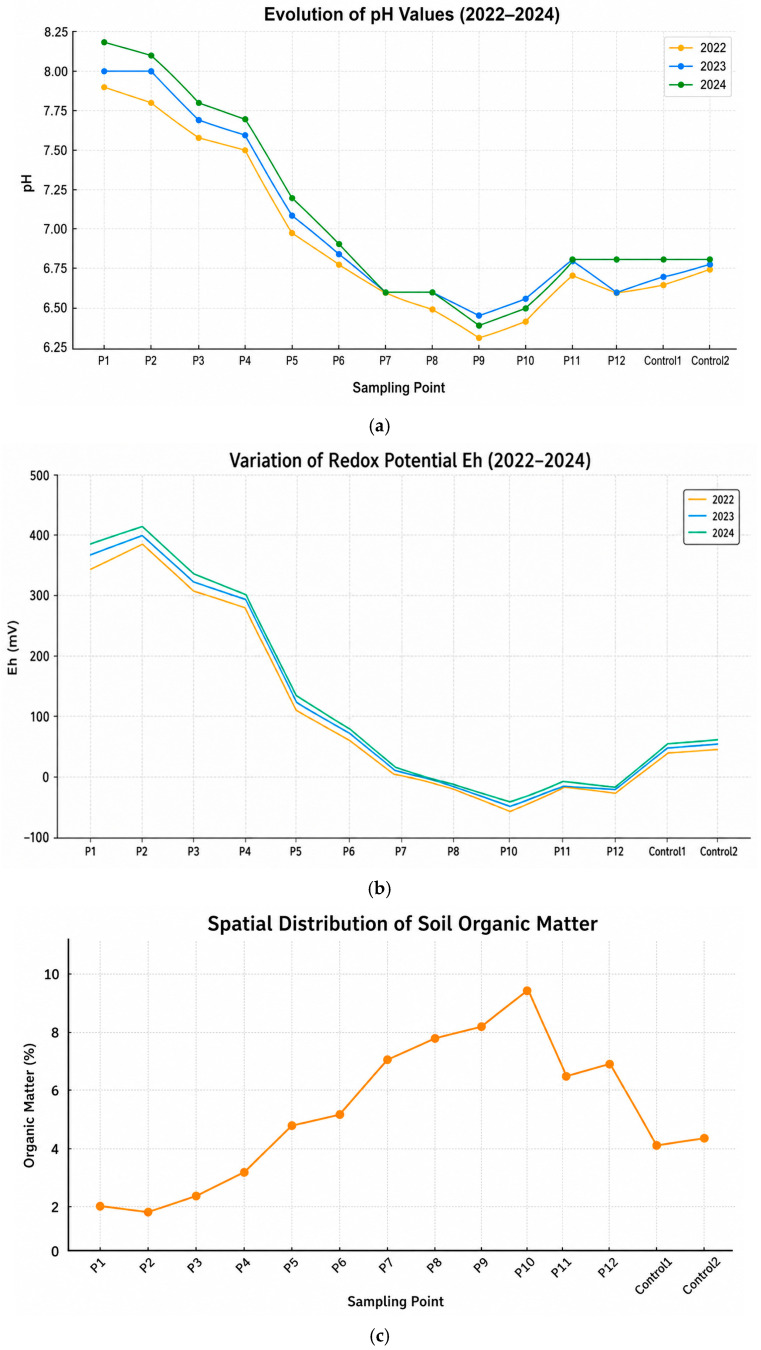
Spatial variation in key physicochemical parameters across the monitored points during 2022–2024: (**a**) soil pH; (**b**) redox potential (Eh); and (**c**) soil organic matter.

The monitoring dataset revealed a pronounced upper-to-lower gradient in pH, redox conditions, moisture, and organic matter (Table 2, Figure 2). The upper sector (P1–P4) remained alkaline throughout the study period, with pH values between 7.5 and 8.2. In contrast, the lower sector (P9–P12) was slightly acidic to near-neutral, with pH values between 6.3 and 6.8. A comparable gradient was observed for Eh. The upper sector showed consistently oxidising field conditions, from +280 to +412 mV, whereas the lower sector showed negative values down to −59 mV, indicating comparatively reducing microenvironments.

Moisture and OM displayed the opposite spatial pattern. The lowest moisture and OM values were found in the upper sector, while the lower sector accumulated water and organic matter, reaching 28.3% moisture and 9.5% OM at P10. Because moisture and OM are tabulated as site descriptors rather than annual inferential variables, they are used to support the redox-zonation interpretation but not to claim statistically tested interannual trends. Together, these patterns define two contrasted geochemical compartments: an upper, aerated and oxidising plateau, and a lower, wetter and comparatively reducing zone, with P5–P8 forming a transition between them.

### 3.2. Distribution of Mn and Fe

**Figure 3:**
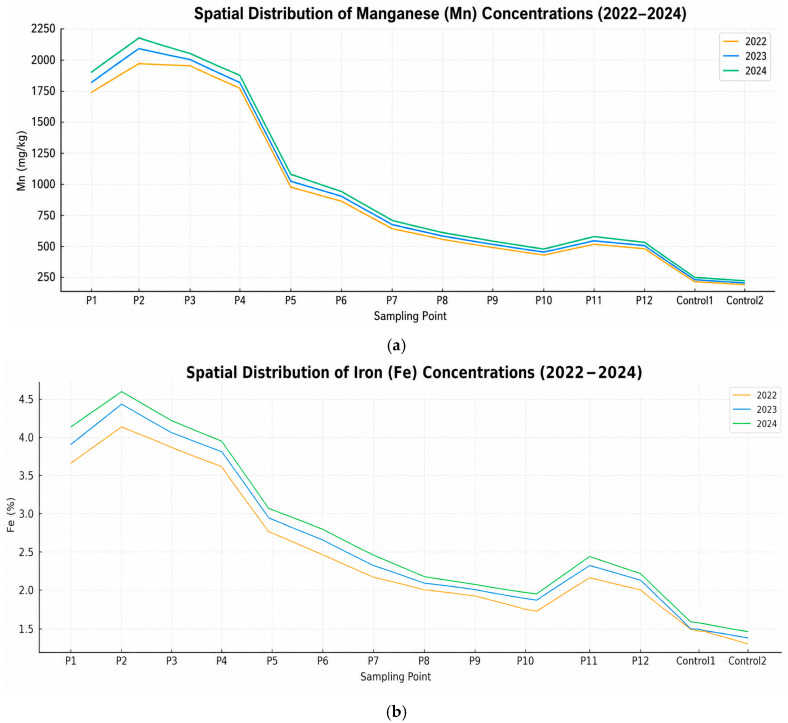
Spatial distribution of redox-active matrix elements during 2022–2024: (**a**) Mn concentrations; (**b**) Fe concentrations.

Mn and Fe concentrations were highest in the upper part of the dump and declined toward the lower sector (Table 3, Figure 3). In 2024, Mn reached 2180 mg/kg at P2 and remained above 1800 mg/kg at several points in the upper plateau, while the two local operational control samples contained only 200–230 mg/kg. Fe followed the same trend, with maxima of 4.6% at P2 and 4.2% at P3. These distributions coincide with the oxidising field conditions identified in the upper sector and are consistent with the presence of redox-active matrix components in the sector where extractable Cr(VI) is highest. However, because mineralogical confirmation was not included, Mn and Fe are interpreted as geochemical indicators rather than direct evidence of specific oxidising mineral phases.

### 3.3. Total Chromium and Extractable Cr(VI)

**Figure 4:**
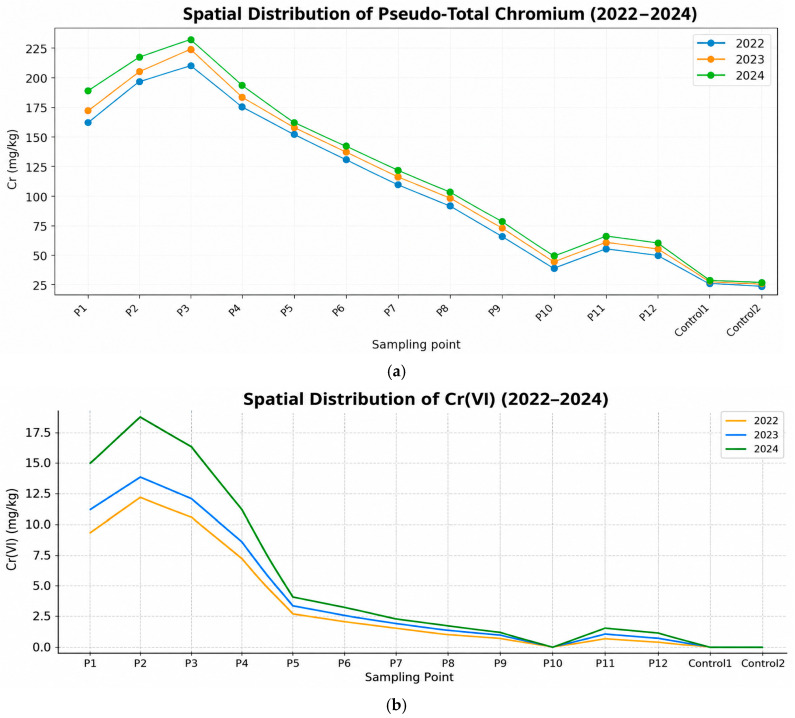
Spatial distribution of chromium species during 2022–2024: (**a**) pseudo-total chromium; (**b**) extractable Cr(VI).

Pseudo-total chromium was elevated throughout the dump relative to the two local operational control samples, but extractable Cr(VI) showed a much sharper spatial contrast than pseudo-total chromium (Table 4, Figure 4). Pseudo-total chromium ranged from 38.2 mg/kg at P10 in 2022 to 231.5 mg/kg at P3 in 2024. By contrast, extractable Cr(VI) was concentrated mainly in the upper sector, where alkaline and oxidising field conditions coincided with elevated Mn and Fe contents. This contrast indicates that chromium-related hazard potential at the site is controlled more by local speciation and redox setting than by pseudo-total chromium concentration alone.

The strongest screening-level toxicological signal was recorded at P2, where extractable Cr(VI) increased from 12.3 mg/kg in 2022 to 18.7 mg/kg in 2024. This 2022–2024 increase should be interpreted as an interannual pattern observed during three late-summer campaigns, not as a statistically demonstrated long-term trend. The campaigns were conducted in late-summer months but not on identical calendar dates; therefore, the observed pattern may reflect a combination of surface oxidation, seasonal moisture differences, and field-scale heterogeneity rather than a strictly linear temporal trajectory. For the present screening objective, the repeated occurrence of the highest values at P1–P3 is more important than the exact year-to-year slope, because it indicates persistence of the same upper-sector hotspot under broadly comparable seasonal monitoring conditions. The upper sector as a whole contained the highest Cr(VI) values, whereas the lower sector remained much lower, and both operational control samples remained below the quantification limit.

The upper-sector values were not borderline detections. In 2024, extractable Cr(VI) at P1–P4 ranged from 11.6 to 18.7 mg/kg, whereas the dry-mass LOQ was approximately 0.12 mg/kg. In contrast, control samples remained below LOQ, and the lowest dump values were close to the lower analytical range. For this reason, mechanistic discussion is centred on the robust upper-sector contrast rather than on small differences among low-concentration points.

### 3.4. Cr(VI)/Pseudo-Total Cr Fraction and R_redox_ Screening

**Figure 5:**
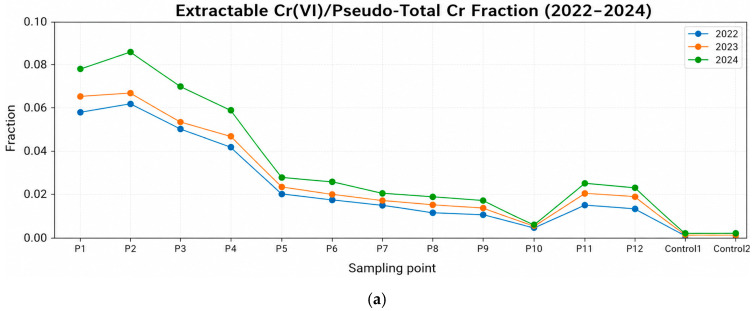

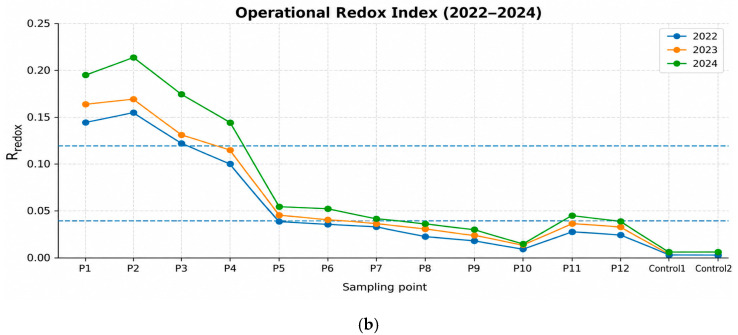
Screening metrics derived from chromium speciation during 2022–2024: (**a**) extractable Cr(VI)/pseudo-total Cr fraction; (**b**) operational R_redox_ values. Blue dashed horizontal lines indicate the operational screening thresholds separating low-priority (R_redox_ < 0.04), moderate-priority (0.04 ≤ R_redox_ < 0.12), and high-priority (R_redox_ ≥ 0.12) classes.

The extractable Cr(VI)/pseudo-total Cr fraction and the associated operational R_redox_ prioritisation score separated the site into high-, moderate-, and low-priority screening sectors (Table 5, Figure 5). The highest fractions were recorded in the upper sector, particularly at P1–P3, where the Cr(VI) fraction exceeded 0.07 in 2024. These points also combined alkaline pH and positive Eh, placing them in the high-priority class according to the internal R_redox_ thresholds.

The operational R_redox_ score reproduced the same spatial structure without introducing a claim of regulatory or toxicological threshold exceedance. In 2024, the score reached 0.215 at P2, 0.195 at P1, 0.175 at P3, and 0.147 at P4, identifying the upper dump sector as the principal internal priority zone for redox-driven extractable Cr(VI) persistence. Values declined sharply downslope, reaching 0.013 at P10 in 2024. Intermediate values at P5–P6 indicate a transition zone rather than a uniformly contaminated sector. This pattern supports the use of R_redox_ as an internal prioritisation layer, while the measured Cr(VI) concentrations remain the primary toxicologically relevant evidence.

### 3.5. Statistical Synthesis

The exploratory statistical synthesis supported the visual and geochemical interpretation of the monitoring dataset (Table 6). Because the same sites were monitored over time, the repeated-site test was used to identify interannual shifts without treating composited subsamples as independent replicates. Significant Friedman test results were obtained for pH, Eh, pseudo-total Cr, and extractable Cr(VI), indicating consistent directional differences across the monitored points within the repeated-site monitoring design.

Correlation analysis highlighted strong coupling between the principal redox-sensitive variables across the pooled monitoring dataset (P1–P12, *n* = 36). The strongest associations were observed between Eh and Cr(VI), Mn and Cr(VI), and Mn and Eh.

The tests are exploratory and are based on repeated monitoring points (P1–P12; *n* = 12 site blocks for Friedman tests and *n* = 36 pooled observations for Spearman correlations); control samples were not included in inferential testing. *p* < 0.05 indicates deviation from normality or a significant repeated-site difference, depending on the column. Because the monitored variables follow the same upper-to-lower geomorphological gradient, the high correlation coefficients should not be interpreted as independent mechanistic evidence of temporal causality or independent process rates. They primarily describe co-located spatial gradients repeatedly observed across the same monitoring points.

Therefore, the correlations support the screening interpretation only when read together with sector-based zonation and direct Cr(VI) measurements.

The sector-based reading led to the same conclusion without relying on correlation strength. Across 2022–2024, the upper sector (P1–P4) combined pH 7.5–8.2, Eh +280 to +412 mV, extractable Cr(VI) 7.3–18.7 mg/kg, and R_redox_ 0.103–0.215. The transitional sector (P5–P8) showed intermediate conditions, with pH 6.5–7.2, Eh −15 to +140 mV, extractable Cr(VI) 1.2–4.4 mg/kg, and R_redox_ 0.023–0.054. The lower sector (P9–P12) remained comparatively reducing, with pH 6.3–6.8, Eh −62 to −10 mV, extractable Cr(VI) 0.24–1.6 mg/kg, and R_redox_ 0.010–0.045.

To support visual assessment of distributional shape and potential outliers, additional boxplots of the main physicochemical and geochemical variables are provided in the Supplementary Materials as Figure S1. Because each reported value represents one site-year composite sample rather than independent laboratory replicates, standard errors were not calculated for individual table entries; reporting artificial error bars would imply a level of replication not present in the sampling design. Descriptive statistics for the main monitored variables are provided in Table S1.

## 4. Discussion

**Figure 6:**
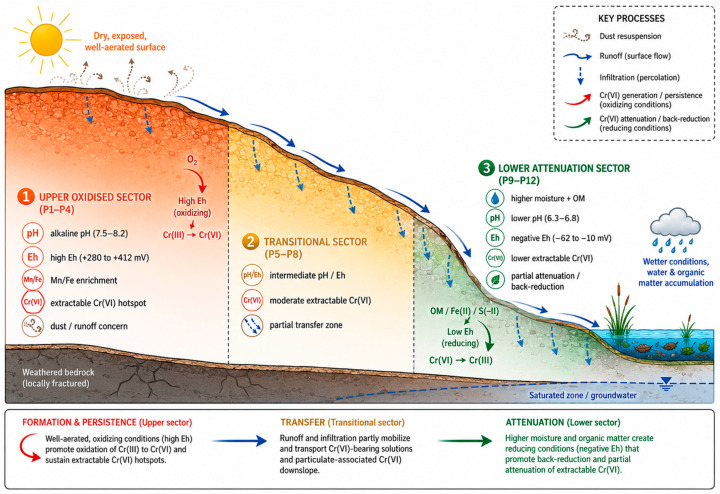
Conceptual model of redox-controlled extractable Cr(VI) hotspot formation, transfer, and attenuation within the Defor Petrila tailings dump.

Figure 6 summarises the conceptual interpretation of redox-controlled extractable Cr(VI) hotspot formation, downslope transfer, and partial attenuation within the Defor Petrila tailings dump. The model integrates the main field observations by distinguishing three functional compartments: an upper oxidised sector where alkaline pH, positive Eh, and Mn/Fe enrichment favour Cr(VI) persistence; a transitional sector where runoff and infiltration may support partial downslope transfer; and a lower, wetter sector where organic matter accumulation and reducing conditions may promote partial attenuation or back-reduction of Cr(VI).

### 4.1. Why the Upper Sector Behaves as a Cr(VI) Hotspot

The Defor Petrila dataset indicates that the most important chromium hazard-screening compartment is not the entire waste body, but a specific redox domain located in the upper sector of the dump. This sector combines the geochemical conditions most favourable to Cr(VI) persistence: alkaline pH, positive Eh, low moisture, low OM, and elevated Mn and Fe concentrations. These observations are consistent with the established mechanism according to which Mn oxides can act as efficient natural oxidants of Cr(III), especially in aerated and alkaline environments [8,11,12,15,16,34]. However, the present dataset demonstrates the field co-occurrence of these conditions and extractable Cr(VI), not the mineralogical identity or reaction kinetics of the oxidising phases.

Therefore, the upper-sector hotspot is best interpreted as a compartment where Cr(VI) production, preservation, and accumulation may act together, rather than as evidence for a single isolated process. The available field data cannot distinguish quantitatively between newly produced Cr(VI), Cr(VI) retained from previous oxidation episodes, and Cr(VI) maintained under favourable alkaline oxidising conditions. Nevertheless, the co-occurrence of elevated extractable Cr(VI), positive Eh, alkaline pH, and Mn/Fe enrichment indicates that the upper sector provides the most favourable geochemical setting for the persistence of the hexavalent form.

This interpretation is further supported by the sector-level contrast and by strong Mn–Eh and Eh–Cr(VI) associations, although these correlations are partly constrained by the spatial structure of the dump and should not be interpreted as independent mechanistic proof. The key implication is that chromium hazard potential is controlled by the interaction between waste composition, redox state, and local geochemical conditions, rather than by pseudo-total chromium alone.

### 4.2. Toxicological Significance and Plausible Exposure Pathways

Cr(VI) is toxicologically important because it is the chromium form most closely associated with mobility, bioavailability, and adverse health effects, including carcinogenicity under relevant exposure conditions [1,2,4,9,20]. At Defor Petrila, the elevated method-defined extractable Cr(VI) concentrations recorded in near-surface oxidised materials indicate that exposure-oriented investigations cannot be dismissed as unnecessary, even though exposure itself was not measured in this study. From a toxicological perspective, the relevance of the upper sector does not derive solely from elevated chromium concentrations, but from the occurrence of chromium in a method-defined extractable hexavalent form within near-surface materials that may be subject to erosion, dust generation, runoff, or infiltration. This does not quantify receptor exposure, but it identifies the sector where exposure-oriented testing would be most scientifically justified.

The upper plateau is an exposed compartment where fine tailings may be subject to erosion and dust resuspension during dry periods, while downslope migration can occur through runoff and infiltration during wet periods. Recent studies on mining- and smelting-impacted dusts and tailings show that particle mobilisation, inhalation bioaccessibility, oral bioaccessibility, leaching behaviour, and mineralogical host phases can substantially influence human-health relevance, even when total metal concentrations alone do not fully explain exposure potential [17,20,21,23,25,26].

At the same time, the present results should be interpreted as first-tier environmental hazard screening rather than as a complete human-health or ecological risk assessment. No direct measurements of airborne dust, leachate, groundwater, plant uptake, bioaccessibility, ecotoxicity, or human biomarkers were included. The study, therefore, identifies credible extractable Cr(VI) hotspots and plausible transfer pathways, but it does not quantify dose, exposure frequency, receptor sensitivity, or receptor-specific risk. This distinction is essential because bioaccessibility and exposure-based risk estimates depend strongly on particle size, mineralogy, simulated body-fluid chemistry, leaching behaviour, and site-specific receptor activity [21,22,23,24,25].

The absence of direct exposure measurements is treated here as a boundary condition, not as an implicit risk estimate. Within a tiered assessment logic, the present manuscript contributes Tier 1 evidence: identification of the locations where the toxic chromium fraction is most likely to persist under field conditions. Tier 2 work should test mobilisation and bioaccessibility through leaching, runoff, dust, and simulated gastrointestinal or lung-fluid extraction assays, whereas Tier 3 work should quantify receptor-specific exposure, ecotoxicological effects, and risk. This framing allows management prioritisation without overstating human-health or ecological risk.

### 4.3. Lower-Sector Attenuation Potential and Its Limits

The lower sector of the dump behaved as a comparatively reducing compartment, with lower pH, negative Eh, higher moisture, and higher OM. Under such conditions, Cr(VI) remained low, and the R_redox_ values were consistently small. This pattern is compatible with partial natural attenuation because organic matter can promote reducing microenvironments, provide electron-donating capacity, and favour the reduction of Cr(VI) to less mobile Cr(III) species. Higher moisture may further limit oxygen diffusion and support reducing conditions in fine-grained or locally saturated materials. This interpretation is consistent with the lower extractable Cr(VI) concentrations measured at P9–P12, but it should not be read as proof of a single Cr(VI)-removal mechanism [14,15,35].

However, the present dataset cannot quantitatively separate reduction, sorption/fixation, dilution by less contaminated material, or limited downslope input of oxidised chromium from the upper sector. For this reason, the lower sector is interpreted as an attenuation-prone compartment rather than as direct evidence of permanent Cr(VI) immobilisation. Its apparent buffering role depends on the persistence of reducing conditions, sufficient moisture retention, and continued organic matter influence.

This attenuation function should, therefore, be considered conditional. If drought, drainage changes, vegetation loss, erosion, internal material redistribution, or decomposition of organic matter shift the lower sector toward more oxidising conditions, previously attenuated chromium could become more environmentally available. In practical terms, the low Cr(VI) values measured at P9–P12 indicate lower current screening priority, not permanent absence of hazard. Monitoring and management should, therefore, treat the dump as a connected redox system in which upper-sector Cr(VI) generation or persistence, transitional transfer, and lower-sector attenuation may vary over time.

### 4.4. Usefulness and Limits of R_redox_ for Hazard-Based Site Screening

The main methodological contribution of this manuscript is the use of an operational, reproducible redox-based prioritisation score as a transparent decision-support layer for Cr(VI) persistence. R_redox_ condensed measured Cr(VI) occurrence and supporting field conditions into a single, spatially interpretable pattern that matched the measured Cr(VI) distribution and the field geochemical zonation. Its value lies not in replacing analytical Cr(VI) data, but in helping to prioritise sampling density, remediation planning, and hotspot-focused surveillance at heterogeneous mine-waste sites [17,21,22,24]. The score is particularly useful where managers must decide which sectors should receive denser sampling, leachability testing, bioaccessibility assessment, mineralogical confirmation, or disturbance-control measures first.

Nevertheless, R_redox_ has clear limitations. We, therefore, do not propose R_redox_ as a universal chromium-risk index; rather, we propose it as a transparent and reproducible screening logic that can be recalibrated for other mine-waste settings if local pH–Eh domains, Cr speciation data, mineralogy, hydrology, climate, and exposure conditions are available. It is semi-quantitative, depends on expert-selected weighting factors, and was calibrated only internally against the Defor Petrila monitoring pattern. It does not replace direct measurements of bioaccessibility, leachability, ecotoxicity, mineralogy, or receptor exposure [21,22,23,25,26]. The proposed low, moderate, and high classes should, therefore, be understood as internal site-prioritisation categories rather than regulatory or toxicological thresholds. Its greatest value lies in identifying where toxic chromium is most likely to remain stable and where additional toxicological investigation is most warranted.

Transfer of the same numerical classes to other tailings dumps would require independent calibration against local mineralogy, hydrology, climate, and exposure scenarios. Accordingly, the manuscript remains valid even if a reader chooses not to use the numerical R_redox_ classes. The measured Cr(VI) distribution, sector-based contrast, and pH-Eh-Mn pattern independently identify the same upper-sector priority zone. The score improves communication and ranking, but it is not required to establish the main field observation.

The sensitivity check further supports this conservative use of the index. When alpha and beta were varied by ±0.1, the identification of the upper dump sector as the dominant Cr(VI) hotspot remained stable, especially for P1–P3 across the monitoring period and for P4 after 2022. Only points located close to class boundaries changed category under the perturbation scenario, indicating that R_redox_ is most reliable for recognising persistent hotspot compartments and should be interpreted cautiously for transitional locations near class thresholds.

Relative to broader previous work performed at the Defor Petrila site [27], the present manuscript narrows the focus to the toxicologically most relevant chromium fraction and to the field conditions that govern its persistence. This shift moves this study beyond descriptive contamination mapping and toward an explicit interpretation of toxic species occurrence, redox-domain hotspot identification, and monitoring prioritisation. The distinction is important for publication in *Toxics* because the manuscript addresses the environmental behaviour of a toxic species and defines where exposure-oriented validation should be targeted next.

### 4.5. Precautionary Management and Monitoring Implications

From a practical management perspective, the results do not imply uniform intervention across the entire tailings dump. Instead, they indicate that monitoring and precautionary control measures should first focus on the upper oxidised sector, where extractable Cr(VI), positive Eh, alkaline pH, Mn enrichment, and elevated R_redox_ scores converge. This sector should be prioritised for denser confirmatory sampling, surface stabilisation, dust-resuspension control, runoff interception, and avoidance of uncontrolled material redistribution during any earthworks or reuse planning. Such measures are justified as precautionary hazard-management actions, pending confirmatory exposure-based assessment, rather than as responses to a quantified receptor-specific risk.

The lower sector requires a different monitoring logic. Although it currently behaves as a comparatively reducing attenuation compartment, this function may be sensitive to drying, erosion, vegetation loss, or changes in drainage. For that reason, management should not treat low-R_redox_ areas as safe by default, but as locations where periodic redox monitoring, pore-water or runoff checks, and disturbance control are needed to verify that attenuation conditions are maintained.

### 4.6. Methodological Constraints and Future Validation Needs

Several constraints should be explicitly recognised. First, pseudo-total Cr, Mn, and Fe were determined after acid digestion and, therefore, represent operationally defined acid-extractable pools rather than complete mineralogical inventories [30]. Second, the Cr(VI) dataset reflects method-defined extractable Cr(VI), and its comparability depends on extraction conditions, pH control, matrix effects, and redox preservation during sample handling [29,32,33]. Third, the available QA/QC information supports screening-level interpretation but does not provide a full interlaboratory validation dataset, matrix-spike recovery series, or certified reference material confirmation for Cr(VI) in this specific tailings matrix. Fourth, the control samples provide local operational reference contrast but do not define a statistically robust regional geochemical baseline. Fifth, the sampling campaigns were conducted in late-summer months but not on identical calendar dates, so the design cannot fully separate interannual evolution from short-term seasonal moisture effects. Sixth, the statistical relationships are influenced by the strong spatial gradient of the dump and should, therefore, be interpreted as exploratory field evidence rather than causal proof. These constraints do not invalidate the hotspot pattern, but they define the appropriate level of inference for the study.

Future work should test the proposed hotspot model through a tiered validation strategy. The first validation tier should include repeated seasonal sampling, leachability assays, pore-water or runoff monitoring, and targeted dust sampling in the upper sector. The second tier should address toxicological relevance through oral and inhalation bioaccessibility, ecotoxicity tests, and plant-uptake assessment. The third tier should confirm the controlling mechanisms using mineralogical and speciation methods such as XRD, SEM-EDS, sequential extraction, or X-ray absorption spectroscopy [21,22,23,24,25]. These additions would convert the present hazard-screening framework into a stronger exposure- and mechanism-based risk assessment.

Overall, these limitations do not create unsupported conclusions because each inference is matched to the level of evidence available. The manuscript demonstrates a robust field-screening pattern for extractable Cr(VI) and redox conditions, while deliberately leaving mineralogical causality, leaching behaviour, bioaccessibility, ecotoxicity, and receptor-specific risk for subsequent validation. This positioning is appropriate for a conservative first-stage assessment of an abandoned mine-waste site.

## 5. Conclusions

The Defor Petrila tailings dump functions as a strongly heterogeneous redox system in which chromium-related hazard potential depends on the spatial control of speciation rather than on pseudo-total Cr alone. In this study, pseudo-total Cr refers to the acid-digestible chromium pool obtained by EPA Method 3051A-type digestion, not to the complete chromium inventory of the mineral matrix. The upper sector of the dump, characterised by alkaline pH, positive redox potential, and elevated Mn and Fe contents, constitutes a persistent priority zone for method-defined extractable Cr(VI), with concentrations reaching 18.7 mg kg^−1^ in 2024. By contrast, the lower sector behaved as a wetter, more reducing compartment with lower Cr(VI) values and lower R_redox_ scores.

This conclusion does not depend exclusively on the R_redox_ score: the same priority sector is identified by direct extractable Cr(VI) concentrations, Cr(VI)/pseudo-total Cr fractions, alkaline and oxidising field conditions, Mn enrichment, and sector-level spatial coherence. R_redox_ is, therefore, best understood as a transparent prioritisation layer placed over a measured geochemical pattern, not as a stand-alone risk metric or receptor-specific assessment tool.

These findings show that abandoned mine wastes can contain internally differentiated redox compartments, even within the same deposit, and that hotspot recognition is essential for meaningful monitoring. The operational R_redox_ score proved useful as a first-stage hazard-prioritisation tool for identifying sectors with elevated potential for Cr(VI) persistence and transfer, especially when interpreted together with measured Cr(VI) and field redox conditions rather than as an independent risk score. Because the present study did not include exposure, bioaccessibility, leachability, ecotoxicity, or receptor-specific measurements, the results should be used to define priorities for confirmatory investigation rather than to quantify human-health or ecological risk.

For site management, the results support targeted prioritisation of the upper oxidised sector for confirmatory testing, surface stabilisation, dust-resuspension control, and runoff management, while the lower sector should remain under periodic surveillance to verify that reducing attenuation conditions persist. Overall, this study demonstrates that integrating field geochemistry with chromium speciation offers a robust and conservative hazard-screening framework for prioritising post-mining sites where the toxic form of chromium may remain environmentally active.

## Figures and Tables

**Table 1:** pH and Eh weighting factors used for the calculation of the operational R_redox_ prioritisation score.

Factor	Class	Score	Interpretation
α(pH)	pH < 6.5	0.8	Less favourable for Cr(VI) persistence
α(pH)	6.5 ≤ pH < 7.5	1.0	Transitional pH condition
α(pH)	pH ≥ 7.5	1.2	Alkaline condition favouring Cr(VI) persistence
β(Eh)	Eh < 0 mV	0.8	Reducing condition
β(Eh)	0 ≤ Eh < 200 mV	1.0	Transitional redox condition
β(Eh)	Eh ≥ 200 mV	1.3	Oxidising condition favouring Cr(VI) persistence

**Table 2:** Physicochemical characteristics of near-surface tailings-affected materials and local operational control samples during the 2022–2024 monitoring period.

Sampling Point	pH 2022	pH 2023	pH 2024	Eh 2022 (mV)	Eh 2023 (mV)	Eh 2024 (mV)	Moisture (%)	OM (%)
P1	7.9	8.0	8.2	+350	+372	+390	6.4	2.0
P2	7.8	8.0	8.1	+390	+402	+412	6.0	1.8
P3	7.6	7.7	7.8	+310	+325	+338	7.5	2.3
P4	7.5	7.6	7.7	+280	+295	+302	8.1	3.2
P5	7.0	7.1	7.2	+120	+130	+140	12.5	4.8
P6	6.8	6.9	6.9	+70	+80	+85	15.4	5.2
P7	6.6	6.6	6.7	+10	+17	+20	21.3	7.1
P8	6.5	6.6	6.6	−15	−10	−12	22.9	7.8
P9	6.3	6.4	6.4	−30	−38	−35	25.6	8.2
P10	6.4	6.5	6.5	−55	−62	−59	28.3	9.5
P11	6.7	6.8	6.8	−20	−15	−10	19.4	6.5
P12	6.6	6.6	6.7	−25	−21	−18	20.8	6.9
Control 1	6.7	6.7	6.8	+45	+50	+55	11.3	4.1
Control 2	6.8	6.8	6.8	+55	+58	+60	10.8	4.3

Note: Moisture and OM are reported as site descriptors for the monitored points and were used to support the redox-zonation interpretation; they were not treated as annual repeated-measures variables in the inferential analysis.

**Table 3:** Acid-extractable Mn and Fe contents in soil samples collected from the Defor Petrila tailings dump during 2022–2024.

Sampling Point	Mn 2022 (mg/kg)	Mn 2023 (mg/kg)	Mn 2024 (mg/kg)	Fe 2022 (%)	Fe 2023 (%)	Fe 2024 (%)
P1	1750	1820	1900	3.7	3.9	4.1
P2	1980	2100	2180	4.2	4.5	4.6
P3	1960	2010	2050	3.9	4.1	4.2
P4	1780	1820	1875	3.7	3.8	3.9
P5	980	1020	1080	2.9	3.0	3.1
P6	860	900	930	2.6	2.7	2.8
P7	640	680	700	2.3	2.4	2.4
P8	550	580	600	2.1	2.1	2.2
P9	480	510	530	2.0	2.0	2.1
P10	420	440	460	1.8	1.9	1.9
P11	510	540	570	2.2	2.3	2.4
P12	480	500	520	2.1	2.2	2.2
Control 1	210	220	230	1.5	1.5	1.6
Control 2	180	190	200	1.4	1.4	1.5

**Table 4:** Pseudo-total chromium and method-defined extractable Cr(VI) concentrations (mg/kg) in near-surface tailings-affected materials from the Defor Petrila dump during 2022–2024.

Sampling Point	Pseudo-Total Cr 2022	Pseudo-Total Cr 2023	Pseudo-Total Cr 2024	Cr(VI) 2022	Cr(VI) 2023	Cr(VI) 2024
P1	162.3	171.8	189.5	9.5	11.2	14.8
P2	198.4	205.2	218.1	12.3	13.8	18.7
P3	212.0	226.3	231.5	10.6	12.1	16.4
P4	175.6	182.9	194.2	7.3	8.5	11.6
P5	152.1	158.4	160.2	3.1	3.8	4.4
P6	130.3	136.7	140.1	2.4	2.8	3.6
P7	108.2	115.6	118.4	1.9	2.1	2.4
P8	92.5	96.1	100.4	1.2	1.5	1.9
P9	68.2	72.3	75.4	0.8	1.0	1.3
P10	38.2	42.8	46.7	0.24	0.28	0.31
P11	55.1	60.3	64.5	0.9	1.3	1.6
P12	48.3	52.5	56.1	0.7	1.0	1.3
Control 1	24.4	26.1	25.2	<LOQ	<LOQ	<LOQ
Control 2	22.1	21.8	23.5	<LOQ	<LOQ	<LOQ

LOQ = limit of quantification.

**Table 5:** Extractable Cr(VI)/pseudo-total Cr fraction and operational R_redox_ values used for internal prioritisation of the Defor Petrila tailings dump.

Point	Fraction 2022	Fraction 2023	Fraction 2024	R_redox_ 2022	R_redox_ 2023	R_redox_ 2024
P1	0.058	0.065	0.078	0.145	0.163	0.195
P2	0.062	0.067	0.086	0.155	0.168	0.215
P3	0.050	0.053	0.070	0.125	0.133	0.175
P4	0.041	0.046	0.059	0.103	0.115	0.147
P5	0.020	0.024	0.027	0.040	0.048	0.054
P6	0.018	0.020	0.026	0.036	0.040	0.052
P7	0.017	0.018	0.020	0.034	0.036	0.040
P8	0.013	0.016	0.019	0.023	0.029	0.034
P9	0.012	0.014	0.017	0.019	0.022	0.027
P10	0.006	0.007	0.007	0.010	0.013	0.013
P11	0.016	0.021	0.025	0.029	0.038	0.045
P12	0.014	0.019	0.023	0.025	0.034	0.041
Control 1	<0.002	<0.002	<0.002	<0.004	<0.004	<0.004
Control 2	<0.002	<0.002	<0.002	<0.004	<0.004	<0.004

**Table 6:** Summary of statistical tests applied to the main variables of the monitoring dataset.

Parameter	Shapiro–Wilk W	Normality *p*	Repeated-Site Test	Test *p*	Main Exploratory Correlation	Spearman rho	Correlation *p*
pH	0.877	<0.001	Friedman	<0.001	pH–Eh	ρ = 0.953	<0.001
Eh	0.825	<0.001	Friedman	0.002	Eh–Cr(VI)	ρ = 0.979	<0.001
Pseudo-total Cr	0.931	0.028	Friedman	<0.001	Pseudo-total Cr–Eh	ρ = 0.945	<0.001
Cr(VI)	0.811	<0.001	Friedman	<0.001	Cr(VI)–Mn	ρ = 0.991	<0.001

## Data Availability

The original contributions presented in this study are included in the article/Supplementary Materials. Further inquiries can be directed to the corresponding author.

## Supplementary Materials

The following supporting information can be downloaded at: https://www.mdpi.com/article/10.3390/toxics14060479/s1. Figure S1: Boxplots showing the distribution of the main physicochemical and geochemical variables; Table S1: Descriptive statistics for the main monitored variables.
