# Supplementary figures and images for: Extractable Cr(VI) Hotspots in the Defor Petrila Tailings Dump, Romania: A Redox-Based Hazard Screening Approach
Source: Toxics. 2026 May 30;14(6):479. doi: 10.3390/toxics14060479 (PMC13307838; doi:10.3390/toxics14060479)

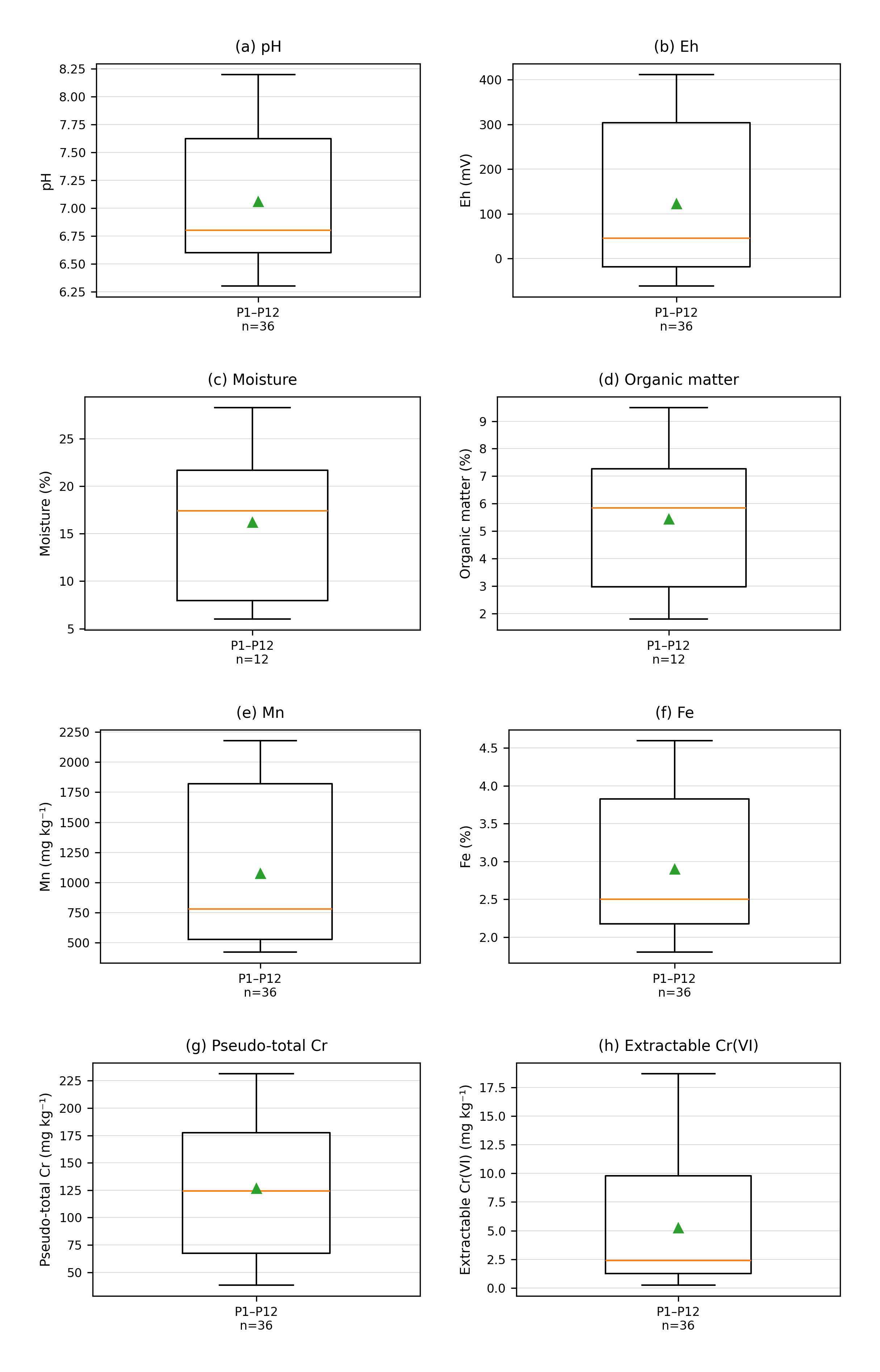

Supplement: Supplementary file 1 [file toxics-14-00479-s001.zip › Figure_S1_boxplots_main_variables.tif]
